# Evaluation of the Color of Zirconia in Different Substrates of Osseointegrated Implants, Thickness of Materials and Types of Resin Cements

**DOI:** 10.1155/2024/8696008

**Published:** 2024-09-27

**Authors:** Aida Seyidaliyeva, Andreas Zenthöfer, Stefan Rues

**Affiliations:** Dental School, Department of Prosthodontics, University of Heidelberg, Im Neuenheimer Feld 400, Heidelberg 69120, Germany

**Keywords:** cement type, color change, surface conditioning of titanium, zirconia thickness

## Abstract

**Objectives:** To evaluate the impact of surface conditioning of titanium, zirconia thickness, and cement type on the final color of zirconia luted to the titanium.

**Methods:** A total of 192 grade 5 titanium specimens with the final dimensions 10 mm × 10 mm × 2 mm were fabricated and subjected to four different surface conditioning including, that is, sandblasting, etching, and anodization. In addition, 192 zirconia specimens with the same dimensions as the titanium specimens but altered thicknesses of 0.7 (*n* = 96) and 1.0 (*n* = 96) mm were fabricated using 5Y-TZP zirconia. Color as expressed by *L*^*∗*^ (lightness), *a*^*∗*^ (red–green axis), and *b*^*∗*^ (blue–yellow axis) of titanium and zirconia specimens as well as the joined titanium–zirconia complex, total assembly (Panavia V5 clear, PC; opaque, PO, each *n* = 96) were determined under standardized conditions using a spectroradiometer (SpectraScan P-650). Color differences were calculated using the *ΔE*_00_ formula. ANOVA supplemented with post hoc Tukey test for group comparisons was compiled to estimate possible effects of titanium conditioning, zirconia thickness, and type of cement used on the final zirconia color (SPSS Ver. 28; *α* = 0.05).

**Results:** All investigated factors affected the zirconia color of the total assembly (*p*  < 0.001). Using PO mean values of all groups were still close to baseline colors (*ΔE*_00_ between 5.5 and 6.2). When using PC, the final color was significantly altered, irrespective of the other parameters. Specimens luted with PO appeared lighter, less reddish (*a*^*∗*^ was affected predominately by sample thickness), and more bluish, while luting with PC resulted in reduced lightness combined with large shifts along the red and yellow axes.

**Significance:** Color changes of zirconia luted to titanium are primarily affected by the color of the substrate if a translucent cement was used. Vice versa, the application of an opaque cement effectively masked the dark substrate color. Substrate color and choice of cement have to be taken into consideration when performing shade selection.

## 1. Introduction

The increasing global usage of dental implants leads to the development of innovative implant materials or modifications. Despite the promising success rate of new materials, titanium and titanium alloys remain the gold standard in dental implant rehabilitation [1]. Nevertheless, there are some disadvantages of titanium and its alloys, particularly in the esthetic zone. The metallic gray hue of the abutment material could cause an unesthetic [2–4]. When it comes to tooth color clinicians evaluate the esthetic outcomes of treatments using the “acceptability threshold (AT)” and “perceptibility threshold (PS).” If the color difference (given by *ΔE*_00_) is below the PS value, the color matching is considered “perfect.” According to the literature (ISO/TR 28642), the International Organization for Standardization has established that the 50% PS is at *ΔE*_00_ = 0.8, and the 50% AT is at *ΔE*_00_ = 1.8 [5, 6].

To ensure desirable outcomes in esthetic rehabilitation, several factors must be considered for implant-supported fixed dental prostheses. These factors include the color and conditioning of titanium abutments, the choice of framework and/or veneering materials, the optical properties of the cements, and the thicknesses of the adhesive layer as well as the restorative materials.

According to literature the abutments with a yellowish or golden hue have a positive impact on the overall esthetics of restorations and soft tissues [7]. Currently, various anodization procedures are employed to alter the color appearance of titanium and its alloys [8–10]. The desired color achieved through anodizing procedures results from a change in the oxide layer thickness which can be controlled by the magnitude and duration of the applied voltage [11–14]. Nevertheless, even if anodization procedures are conducted under identical conditions (same voltage and application time), the final color of titanium specimens can significantly differ due to variations in surface roughness [15].

Due to their superior esthetic and physical properties when compared to dental composites, the utilization of dental ceramics has gained prominence as veneering materials or metal-free restorations [16–18]. Silicate and glass ceramics have superb esthetic properties but are lacking with regard to tensile strength, thus requiring rather high wall thicknesses. Tetragonal zirconia polycrystal ceramics, doped with 3 mol% yttria (3Y-TZP), are known for their exceptional physical properties (with bending strength values of 900–1200 MPa and fracture toughness of 5–10 MPam^0.5^ but inferior esthetics with regard to glass ceramics. They are particularly utilized in short- and long-span fixed dental prostheses [19]. By doping the material >3 mol% yttria, the zirconia ceramics will contain a cubic phase with better esthetic properties besides the tetragonal crystals. Dotage with 7 mol% yttria completely stabilizes the cubic phase, subsequently, 5 mol% (5Y-TZP) leads to each 50% tetragonal and cubic phase. Thus, a dental technician can nowadays choose from many types of zirconia ceramics [20, 21]. Highly translucent and esthetically enhanced zirconia, which is, in general, stronger and more reliable than glass ceramics [22], enabled the use of partially veneered and monolithic zirconia restorations in the anterior area.

Highly translucent ceramics, however, allow the color of a dark substrate to shine through, resulting in esthetic drawbacks in the anterior tooth area, especially when using titanium abutments. To achieve a more pleasant appearance, the coloring of titanium abutments can be performed by anodization. With such a colored titanium abutment, a translucent adhesive will be used. In contrast, opaque cements are often combined with noncolored titanium abutments with the intention to mask them. Other factors that influence the final color of the restoration include the thickness of the material and the cement film thickness and color [23, 24].

Consequently, zirconia ceramics with high yttria content are employed increasingly in modern esthetic dentistry. Moreover, the masking ability of these materials in different thickness and color of cements is a crucial factor, particularly in implant-supported fixed prostheses. Literature is lacking systematic studies on the impact of titanium condition and variation of restoration-related factors such as wall thickness on the final color outcome.

This in vitro study aimed to explore the impact of differently conditioned titanium grade 5 substrates, zirconia wall thickness, and cement type on the final color of the zirconia specimens. The null hypothesis stated that the aforementioned factors do not affect the final coloration of the zirconia specimens.

## 2. Materials and Methods

### 2.1. Sampling

**Figure 1:**
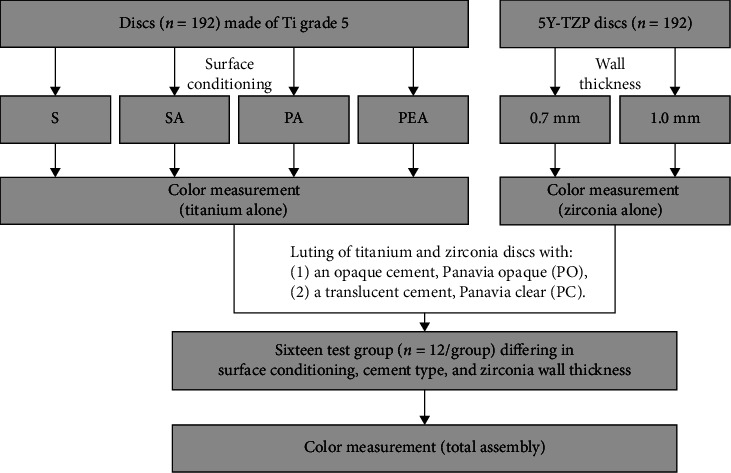
Overview of the study´s schedule and the different test groups. Abbreviations used for different surface treatments: S, sandblasting; SA, sandblasting and anodization; PA, polishing and anodization; PEA, polishing, etching, and anodization.

A total 192 substrate specimens were fabricated from medical titanium grade 5 alloy (Titanit 5, Ti-6Al-4V ELI; Zirkonzahn, South Tyrol, Italy) and divided into four groups (*n* = 48/group) differing in surface conditioning: sandblasting (S), sandblasting + anodizing (SA), polishing + anodizing (PA), and polishing + acid etching + anodizing (PEA). In total, 192 zirconia specimens with thicknesses of 0.7 mm (*n* = 96) and 1.0 mm (*n* = 96) were fabricated from highly translucent zirconia with 5 mol% yttria content (VITA YZ XT, Shade A3; Vita Zahnfabrik, Bad-Säckingen, Germany). Panavia V5 (Kuraray, Chiyoda, Japan) clear (PC) (*n* = 96) and opaque (PO) (*n* = 96) were used as cement. The filler content (38 vol%) is the same for both cement types. They differ in the pigmentation of the resin matrix which is either translucent (Panavia V5 clear) or white (Panavia V5 opaque). An overview of the workflow and the test groups of this study is presented in Figure 1. Since there was no reliable data for a sample size calculation, *n* = 12 was chosen as sample size of the subgroups of this explorative study. With this sample size, group differences exceeding the PS can be detected as long as the standard deviation within the groups is less than 80% of the PS.

### 2.2. Fabrication and Surface Conditioning of the Titanium Substrates

The process of surface preparation of titanium specimens involved the utilization of a semiautomatic grinding and polishing device (Tegramin 25; Struers, Willich, Germany). To achieve the planar and homogenous surface on the specimens, planar grinding with diamond discs (MD Piano #220; Struers) was performed.

The final dimensions (10 × 10 × 2 ± 0.2 mm) of all specimens were checked and recorded utilizing a digital micrometer screw (Micromar 40 EWR, Mahr, Göttingen).

The substrate specimens were categorized into four main groups (*n* = 48/group). The groups differed in the surface conditioning techniques applied to the substrate specimens: S, SA, PA, PEA. A detailed description of surface conditioning steps is provided in Table 1.

### 2.3. Fabrication of the Zirconia Discs

For the fabrication of zirconia specimens with nominal thicknesses of either 0.7 mm or 1.0 mm, blanks made of highly translucent zirconia with 5 mol% yttria content (VITA YZ XT, Shade A3; Vita Zahnfabrik, Bad-Säckingen, Germany) were utilized. Blocks with quadratic cross-section were cut from the blanks and sectioned afterward to produce zirconia discs (Isomet High Speed Pro, Buehler, Lake Bluff, Illinois, USA). Disc dimensions were chosen such that dimensions after sintering according to the manufacturer's instructions (Programat S1 1600, Ivoclar Vivadent AG, Lichtenstein) were 10 mm × 10 mm × 1.0 mm/1.3 mm (*n* = 96 for each thickness). Access material in the vertical direction was removed during grinding and prepolishing processes with diamond discs followed by a two high gloss polishing steps with diamond suspension (identical to the polishing procedure described in Table 1 for the titanium samples).

After cleansing the specimens in an ultrasonic cleaner with distilled water, the final dimensions of the zirconia discs were measured with a digital micrometer screw (Micromar 40 EWR, Mahr, Göttingen, Germany). Dimensions did not deviate more than 20 *μ*m in horizontal and vertical direction from the nominal values.

### 2.4. Luting of Zirconia and Titanium Specimens

To adhesively attach the zirconia specimens to the titanium counterparts, Panavia V5 (Kuraray, Chiyoda, Japan) clear (PC) and opaque (PO) were employed, resulting in a total of 96 pairs for each type. As a pretreatment method for the zirconia specimens, a tribochemical silica coating was employed (Rocatec; 3M, Seefeld, Germany) on the surface to be bonded. The process involved sandblasting with Rocatec Pre at a distance of 10 mm for 10 s at a 45° angle and 0.28 MPa air pressure, followed by sandblasting with Rocatec Plus in the same manner [25].

Following the manufacturer's recommendations, Clearfil Ceramic Primer Plus (Kuraray, Chiyoda, Japan) was applied to the surfaces of titanium and zirconia specimens. Once the primer was dry, the luting procedure was conducted within a universal testing device (Z005, Zwick/Roell, Ulm, Germany) to achieve a nominal cementation gap of 100 *μ*m. The titanium discs were placed on a sample holder fixated at the lower end of the testing device, whereas the zirconia samples were attached to the crosshead. With each titanium/zirconia disc pair the vertical height at which contact occurred between the discs was determined and set to *z* = 0 mm (testexpert III; ZwickRoell, Germany). After the application of adhesive resin cement, the crosshead was lowered to the vertical position *z* = 0.100 mm and held constant for 14 min to ensure a complete curing of the resin cement at room temperature before sample removal.

The exact final thickness of the luted specimens was verified again with a digital micrometer screw (Micromar 40 EWR, Mahr, Göttingen). Knowing the heights of the respective titanium and zirconia discs, the actual cement gap width could be calculated. Only specimens with a cementation gap width deviating <20 *μ*m from the nominal value (100 µm) were included in the study. Samples that had to be excluded were reproduced until they met inclusion criteria.

### 2.5. Color Measurements

**Figure 2:**
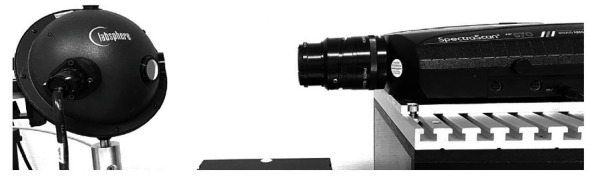
Configuration of the color measurement process for the specimens using a spectroradiometer (SR, SpectraScan PR-650, Photo Research, Chatsworth, CA, USA).

A spectroradiometer (SR, SpectraScan PR-650, MS-75 lens, Photo Research Inc., Chatsworth, California) was used to measure the color of specimens (SR, SpectraScan PR-650, MS-75 lens, Photo Research Inc. Chatsworth, CA, USA) (Figure 2). A 35W tungsten lamp was connected to a stable voltage power supply (IHLS 100-35, Labsphere Inc., North Sutton, NH, USA). An Ulbricht sphere (Labsphere Inc., North Sutton, NH, USA) was integrated to achieve diffuse reflection-free lighting. The aperture of the spectroradiometer was set to 1°. Specimens were placed at a distance of 40 cm from the spectroradiometer using a CAD/CAM fabricated holder. Prior to conducting all measurements, calibration was performed using a white diffuse-reflectance standard (*L*^*∗*^ = 100 and *a*^*∗*^ = *b*^*∗*^ = 0, Spectralon, the world's whitest material). No other light sources were present in the room during the measurements.

The measurements were conducted to determine the *L*^*∗*^ (lightness axis), *a*^*∗*^ (green–red axis), and *b*^*∗*^ (blue–yellow axis) values in the CIELAB color space, based on the standards set by the Commission Internationale de l'Éclairage (CIE), using a 2° observer angle [26]. The recorded *L*^*∗*^, *a*^*∗*^, and *b*^*∗*^ values of the specimens represented the mean values obtained from three measurement repetitions.

Prior to the measurements, all specimens underwent a cleaning process involving distilled water and subsequent air drying. Before the luting process, baseline *L*^*∗*^, *a*^*∗*^, and *b*^*∗*^ values for the zirconia and titanium discs alone were administered first. Titanium discs could be placed in a standard sample holder whereas for the zirconia discs a special sample holder containing a zirconia cube (edge length 12 mm, polished, made of the same zirconia material as the discs) was used. Glycerin was applied to the zirconia block surface and the zirconia discs were placed on top. The measurement results for the titanium substrates were already reported elsewhere [15]. After luting of zirconia discs to titanium substrates, color measurements provided the *L*^*∗*^, *a*^*∗*^, and *b*^*∗*^ values of the total assembly.

Color differences *ΔE*_00_ (titanium/zirconia) between titanium and zirconia samples as well as *ΔE*_00_ (total assembly/zirconia) between zirconia samples and the total assembly were calculated using the CIEDE2000 [27]:

  $$\begin{array}{c} \Delta E_{00}={\left[{\left(\frac{\Delta L^{\prime }}{K_{L}S_{L}}\right)}^{2}+{\left(\frac{\Delta C^{\prime }}{K_{C}S_{C}}\right)}^{2}+{\left(\frac{\Delta H^{\prime }}{K_{H}S_{H}}\right)}^{2}+R_{T}\left(\frac{\Delta C^{\prime }}{K_{C}S_{C}}\right)\left(\frac{\Delta H^{\prime }}{K_{H}S_{H}}\right)\right]}^{\frac{1}{2}}, \end{array}$$

where *K*_*L*_ = *K*_*C*_ = *K*_*H*_ = 1(parametric factors, default values) and:

  $$\begin{array}{c} L_{1/2}^{\prime }=L_{1/2}^{*},\Delta L^{\prime }=L_{2}^{\prime }-L_{1}^{\prime },\overset{―}{L}=\left(L_{1}^{\prime }+L_{2}^{\prime }\right)/2, \end{array}$$

  $$\begin{array}{c} C_{1/2}^{*}=\sqrt{{a_{1/2}^{*}}^{2}+{b_{1/2}^{*}}^{2}},\;{\overset{―}{C}}^{*}=\left(C_{1}^{*}+C_{2}^{*}\right)/2, \end{array}$$

  $$\begin{array}{c} a_{1/2}^{\prime }=a_{1/2}^{*}+\frac{a_{1/2}^{*}}{2}\left(1-\frac{1}{2}\sqrt{\frac{{{\overset{―}{C}}^{*}}^{7}}{{{\overset{―}{C}}^{*}}^{7}+25^{7}}}\right),b_{1/2}^{\prime }=b_{1/2}^{*}, \end{array}$$

  $$\begin{array}{c} C_{1/2}^{\prime }=\sqrt{{a_{1/2}^{\prime }}^{2}+{b_{1/2}^{\prime }}^{2}},\;\Delta C^{\prime }=C_{2}^{\prime }-C_{1}^{\prime },\;{\overset{―}{C}}^{\prime }=\left(C_{1}^{\prime }+C_{2}^{\prime }\right)/2, \end{array}$$

  $$\begin{array}{c} h_{1/2}^{\prime }={\text{Tan}}^{-1}\left(b_{1/2}^{\prime }/a_{1/2}^{\prime }\right)\text{ with }h_{1/2}^{\prime }\in \left[0{}^{\circ},360{}^{\circ}\right], \end{array}$$

  $$\begin{array}{c} \Delta h^{\prime }=h_{2}^{\prime }-h_{1}^{\prime }\text{ with }\Delta h^{\prime }\in \left[-180{}^{\circ},180{}^{\circ}\right], \end{array}$$

(  ) $$\begin{array}{c} \Delta H^{\prime }=2\sqrt{C_{1}^{\prime }C_{2}^{\prime }}\sin\left(\Delta h^{\prime }/2\right),{\overset{―}{H}}^{\prime }=\left\{\begin{array}{c} \left(h_{1}^{\prime }+h_{2}^{\prime }\right)/2 \end{array}\right.\begin{array}{c} \left|\left(\;\right.h_{1}^{\prime }-h_{2}^{\prime }\right|\leq 180{}^{\circ} \end{array}, \end{array}$$

  $$\begin{array}{c} T=1-0.17\cos\left({\overset{―}{H}}^{\prime }-30{}^{\circ}\right)+0.24\cos\left(2{\overset{―}{H}}^{\prime }\right)+0.32\cos\left(3{\overset{―}{H}}^{\prime }+6{}^{\circ}\right)-0.20\cos\left(4{\overset{―}{H}}^{\prime }-63{}^{\circ}\right), \end{array}$$

  $$\begin{array}{c} S_{L}=1+\frac{0.015\left(\overset{―}{L}-50\right)}{\sqrt{20+{\left(\overset{―}{L}-50\right)}^{2}}},S_{C}=1+0.045{\overset{―}{C}}^{\prime },S_{H}=1+0.015{\overset{―}{C}}^{\prime }T, \end{array}$$

  $$\begin{array}{c} R_{T}=-2\sqrt{\frac{{\overset{―}{C}}^{\prime 7}}{{\overset{―}{C}}^{\prime 7}+25^{7}}}\sin\left\{60{}^{\circ}\cdot \exp\left[-{\left(\frac{{\overset{―}{H}}^{\prime }-275{}^{\circ}}{25{}^{\circ}}\right)}^{2}\right]\right\}. \end{array}$$

### 2.6. Statistical Analysis

The statistical analysis was carried out using the software package SPSS, version 28 (IBM, Armonk, NY, USA). In descriptive data analysis, the mean values and standard deviations for ∆*L*^*∗*^, ∆*a*^*∗*^, and ∆*b*^*∗*^ (both states separately) as well as for ∆*E*_00_ (zirconia/total assembly) were computed for each test group. After verifying the normal distribution of our data via Shapiro Wilk tests and QQ-plot inspection, the determination of the effect of each factor (surface conditioning, cement type, zirconia wall thickness) on the dependent variable *ΔE*_00_ was carried out with the aid of ANOVA. Post hoc Tukey tests were used for intergroup comparisons. In addition, the influence of the substrate color on the color of the total assembly was evaluated with linear regression between the states (zirconia/total assembly) and (titanium/zirconia) for ∆*L*^*∗*^, ∆*a*^*∗*^, ∆*b*^*∗*^, and *ΔE*_00_.

## 3. Results

**Figure 3:**
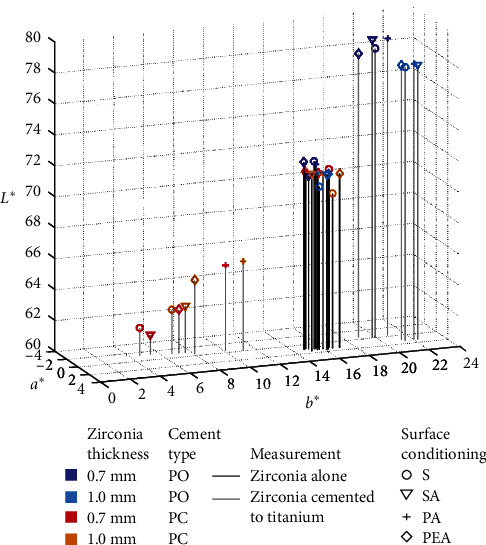
Graph showing the mean *L*^*∗*^*a*^*∗*^*b*^*∗*^ color coordinates of all test groups, both for the baseline measurement (zirconia alone) and the final assembly with zirconia discs cemented to titanium.

The results of the descriptive data analysis showed that the specimens luted with opaque cement (PO) exhibited higher lightness (*L*^*∗*^) and a more yellowish (*b*^*∗*^) color compared to the specimens from the same group luted with clear cement (PC) (Table 2, Figure 3). There was no noticeable difference Δa^*∗*^ between the PO and PC groups along the red–green axis. The maximum relative color difference ∆*E*_00_ (zirconia/total assembly) was observed for specimens of group S-0.7 mm-PC (∆*E*_00_ = 11.7 ± 0.3, ΔL^*∗*^ = -9.70 ± 0.5, and Δb^*∗*^ = -11.8 ± 0.7) and in specimens of group SA-0.7 mm-PC (*∆E*_00_ = 10.9 ± 0.3, ΔL^*∗*^ = -11.0 ± 0.7, and Δb^*∗*^ = 9.6 ± 0.5). The smallest mean relative color difference was noted in the samples of group PEA-0.7 mm-PO (∆*E*_00_ = 5.5 ± 0.4) and PA-1.0 mm-PC (∆*E*_00_ = 5.5 ± 0.3). *ΔE*_00_ values for all groups significantly exceeded the PT (*ΔE*_00_ = 0.8) and AT (*ΔE*_00_ = 1.8) values. All factors (surface conditioning, zirconia thickness, and cement type) and all factor combinations had a significant effect (all *p*-values: *p*  < 0.001) on the color change of zirconia when cemented to a titanium substrate. Since the absolute color differences due to changes in zirconia thickness were rather small, homogeneous subsets were calculated only considering the factors surface conditioning and cement type (Table 3). With opaque cement (PO), mean values of all groups were rather close (*ΔE*_00_ between 5.5 and 6.2), the only significant differences were found between PEA and PA and PEA and SA. In contrast, all test groups differed significantly when translucent cement was used.

**Figure 4:**
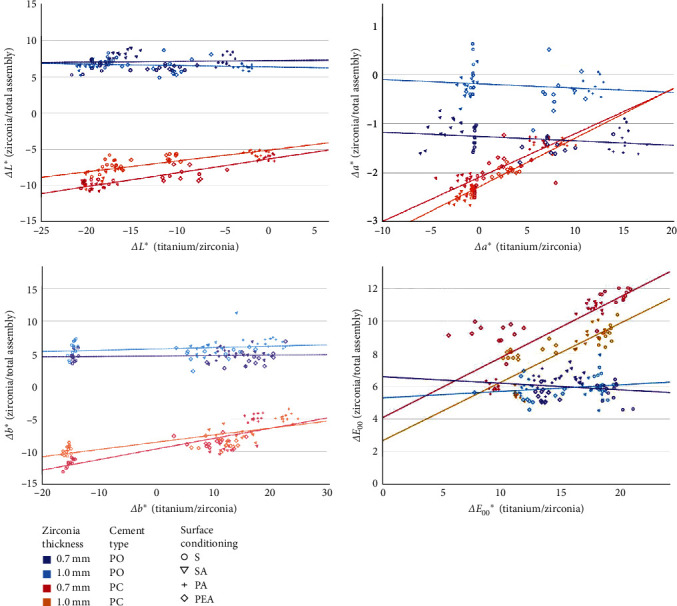
Graphical description of the results of the linear regression analysis.

To analyze how the color coordinate differences (ΔL^*∗*^, Δa^*∗*^, and Δb^*∗*^) as well as color difference *ΔE*_00_ between zirconia and titanium substrate affected the respective values for final samples when compared to zirconia samples before luting, scatter plots of the data were made and linear regression analyses carried out. Results (Table 4, Figure 4) revealed that the color change of the total assembly was only affected by the substrate's color with translucent cement (PO: *p* ≥ 0.065 for all color measures and zirconia thicknesses, PC: *p*  < 0.001 for all color measures and zirconia thicknesses). PC all color coordinates were only slightly affected with regard to the two investigated zirconia thicknesses.

In total, it can be clearly seen that zirconia samples cemented with PO appeared lighter, less reddish, more bluish, and zirconia discs cemented PC were darker and had large shifts along the red and yellow axes. With a 100 µm PO layer between titanium and zirconia, Δa^*∗*^ was affected the most by zirconia thickness and the substrate had no influence. With PC, in contrast, measured color coordinate differences were similar with regard to zirconia thickness, but the substrate color did influence the appearance of the whole assembly.

## 4. Discussion

The study was conducted to determine the possible effects on the final color of a high translucent monolithic zirconia at two different thicknesses, when luted with clear and opaque cements onto titanium substrates that were modified using distinct techniques. It was found that each factor (surface conditioning of titanium, zirconia thickness, and cement type) had a significant effect on the final color of specimens, thus the null hypotheses have to be rejected.

However, different surface conditioning techniques and the thickness of highly translucent ZrO_2_ specimens did not have a significant influence on the final color (*ΔE*_00_ given in Figure 4) of specimens when luted with opaque cement. Furthermore, the color difference between the baseline and final measurements exceeded the PT and AT values, indicating that color alterations were neither perceptible nor acceptable to the observer's eyes. Since the use of PO prevented any influence of the substrate on the color appearance of the total assembly in this study, zirconia anodization is only of interest in combination with translucent cements. Whereas, compared to the color coordinates of zirconia alone, the use of PO led to an increase predominantly in *L*^*∗*^ and *b*^*∗*^, a more or less pronounced decrease in *L*^*∗*^ and *b*^*∗*^ was observed depending on surface conditioning and anodization of the substrate (Figure 3).

In the literature, the color change of ceramic materials attached to titanium substrates has been investigated by various authors. One of them reported that the presence of a titanium substrate has a significant influence on the final color of thin zirconia with thicknesses of 0.5 mm [28].

Similar results regarding an influence of titanium abutments on unacceptable final color were detected in another study. Due to ceramic materials, completely masking the dark color of titanium abutments was not possible. The use of opaque cement was helpful in achieving the desired esthetic appearance as reported by other authors [29].

The outcomes of one study examining the masking efficacy of ceramic discs in conjunction with opaque cement reveal that specimens with thicknesses of 1 and 1.5 mm exhibit a more pronounced masking effect on a dark substrate. The study also validates that the utilization of opaque cement leads to superior concealment of the dark substrate's color compared to translucent cement [24].

Still others concluded that the substrate's color has a diminished impact on the ultimate color when the thickness is 1.5 mm, in addition. Nonetheless, whether utilizing monolithic or layered zirconia at a thickness of 1 mm, the ability to mask effectively is compromised when transparent cement is applied [23]. These findings of both studies align with the results of our own study.

The findings of the study conducted by de Azevedo Cubas et al. [30] show-up with similar results. Their research indicates that both the cement color and the thickness of the ceramic material hold substantial significance with respect to the final color outcome. Specifically, the use of an opaque cement leads to an increase in the *L*^*∗*^, *a*^*∗*^, and *b*^*∗*^ values within the CIELAB color space, regardless of the thickness of the ceramic specimens. This suggests that the choice of cement and ceramic thickness plays a significant role in influencing the resulting color characteristics as elaborated in a previous investigation [30].

### 4.1. Strengths and Limitations

The strength of this study was its meticulous adherence to ISO norms and manufacturers' recommendations throughout all stages of preparation of specimens, surface conditioning, luting, and measurement processes. Furthermore, the study incorporated supplementary standards as advised in the relevant literature, further enhancing its methodological robustness. The specimens were precisely cut from the blanks using a tabletop precision saw (Isomet High-Speed Pro, Buehler, Lake Bluff, Illinois, USA). A semiautomatic grinding and polishing device (Tegramin 25; Struers, Willich, Germany) was utilized to achieve reproducible surface preparation of the titanium specimens. The nominal cementation gap was attained through the use of a universal testing device (Z005, Zwick/Roell, Germany).

Using a spectroradiometer device with an integrated Ulbricht sphere is one of the strengths of the study. As indicated in the literature, spectroradiometers outperform spectrophotometers in achieving more accurate color determination outcomes [31].

There are various approaches available for the evaluation and comparison of the optical appearance or restorative materials, including the translucency parameter (TP), contrast ratio (CR), and color difference equation (*ΔE*_00_).

Creating a natural-looking restoration requires not only color matching (including value, hue, and chroma) but also ensuring the appropriate translucency. In tests with 1 mm thick translucent material samples placed on white and black backgrounds, CR of luminous reflectance (CR = 0: completely translucent, CR = 1: completely opaque) was used to compare dental ceramics. The CR range given for glass ceramics [32] and zirconia [33] was between about 0.3 and 0.7. In particular, among zirconia materials differing in yttria content, CR varied significantly, with the zirconia material used in this study (VITA YZ-XT) at the lower end and the classic 3Y-TZP (VITA YZ-HT) at the upper end of the range mentioned above. Of course, overall translucency of samples made of the same material will decrease with increasing sample thickness [33].

Another strength of our study was to determine the color difference using color difference equation (*ΔE*_00_). Evaluating the relative translucency is feasible through TP, but it does not allow for assessing the influence of substrate color or cement color [30, 34]. In contrast, CR provides insights into material opacity but may not effectively detect minor changes in light transmission for materials with high scattering and absorption coefficients [35–38].

In accordance with the recommendations from CIE, our study made the deliberate choice to employ the CIEDE2000 formulas for the evaluation of color-changing difference.

The utilization of rectangular specimens posed a limitation in our study, as they may not accurately represent the clinical scenario. Another limitation was performing measurements at a single spot with 1° aperture (the midpoint of specimens), disregarding the varying thickness of veneering material across the cervical, middle, and incisal areas in a clinical situation. We opted for noncrown-shaped specimens because utilizing disc-shaped specimens enabled better standardization with regard to cement layer thickness, zirconia wall thickness, and surface conditioning. Additionally, as found by other investigators [39, 40], flat sample surfaces allow for a more precise use of the spectrophotometer and thus more accurate color measurements. The investigated cement gap of 100 µm is representative for the fit between an individual titanium abutment and a zirconia crown, but may be too large with respect to the typical cement gap found between a titanium adhesive base and a zirconia abutment crown. In our study, the opaque cement completely masked the substrates since all PO test groups showed a similar color change. This study cannot answer the question at which point this masking ability is lost when the cement gap width decreases. The investigated zirconia wall thicknesses resemble the minimum wall thickness for 5Y-PSZ (0.7 mm) and a typical wall thickness found on the occlusal surface (1.0 mm). At the margins of dental restorations, zirconia wall thicknesses will decrease to 0.1–0.3 mm.

We selected highly translucent zirconia which the manufacturer primarily indicates for use in the anterior tooth region. Our study focused solely on assessing the material's masking capability on a titanium surface. Our investigation did not encompass color changes of specimens cemented onto natural or tinted tooth surfaces, nor did it extend to zirconia abutments, which are commonly utilized in the anterior tooth area. Titanium and its alloys are still considered as the gold standard for dental implant rehabilitation [1]. However, employing titanium abutments in the anterior region presents challenges for dentists striving to achieve satisfactory esthetic outcomes [2–4]. Hence, our aim was to conduct specimen testing on titanium abutments to tackle this issue.

## 5. Conclusions

Within the limitations of this laboratory study, color change of the overall assembly was primarily influenced by the color of the substrate when translucent cement was used. Furthermore, the application of an opaque cement effectively masked the dark substrate color, regardless of the thickness of the zirconia specimens. Zirconia luted with opaque cement exhibited a lighter appearance, reduced reddish color, and a more pronounced bluish color while the usage of clear cement leads to darker, more reddish, and yellowish color. Clinical studies are encouraged to prove the laboratory outcome.

## 6. Clinical Relevance

This study should help to dental practitioners in their decision-making process regarding the selection of surface conditioning methodologies for titanium abutments, cement type, and the appropriate thickness of veneering material to achieve optimal esthetic outcomes in treatment procedures.

## Figures and Tables

**Table 1:** Details about the different surface conditioning methods used with the titanium alloy specimens.

Surface conditioning step	Description
Sandblasting (S)	Exposition to Al_2_O_3_ particles (50 µm, 2 bar, 5 s, approx. 1 cm distance)
Polishing (P)	Semiautomatic grinding and polishing machine (Tegramin 25; Struers). Pre-polishing: diamond discs (MD Piano #500, MD Piano #1200; Struers) Final polishing: diamond grains (3 µm, 1 µm, DiaPro; Struers) and polishing cloths (MD Nap; Struers)
Etching (E)	Etching solution: 10 g etching powder (sodium bisulfate [NaHSO_4_] and sodium fluoride [NaF]; Zirkonzahn, South Tyrol, Italy) dissolved in 100 ml distilled water Etching procedure: Swinging of specimens in the etching solution for 10 s, followed by rinsing of the specimens with distilled water. Etching and rinsing were carried out twice.
Anodization (A)	Titanium Spectral-Coloring Anodizer (Zirkonzahn) Anodizing solution: 5 g anodizing salt (ammonium sulfate [(NH_4_)_2_SO_4_]; Zirkonzahn, South Tyrol, Italy) dissolved in 250 ml distilled water. Anodizing procedure: The cathode was connected to the negative pole and the titanium specimens to the positive pole of a power supply and submerged in the anodizing solution. A voltage of 60 V was applied for 5 s, which should, according to manufacturer's information, result in a yellow-gold color of the titanium alloy specimen.

**Table 2:** Mean difference of *L*^*∗*^, *a*^*∗*^, and *b*^*∗*^ values and color difference (*ΔE*_00_) between the color of the total assembly compared with zirconia samples alone summarized for all test groups.

Group			Comparison zirconia/total assembly, mean value (SD)			
Surface conditioning	Cement type	Zirconia thickness (mm)	Δ**L**^**∗**^	Δ**a**^**∗**^	Δ**b**^**∗**^	Δ**E**_00_
S	PO	0.7	6.5 (0.7)	−1.3 (0.2)	4.6 (0.7)	5.6 (0.6)
		1.0	7.2 (0.6)	−0.1 (0.4)	5.7 (1.2)	6.2 (0.6)
	PC	0.7	−9.7 (0.5)	−2.3 (0.1)	−11.8 (0.7)	11.7 (0.3)
		1.0	−7.2 (0.9)	- 2.4 (0.1)	−9.8 (0.7)	9.2 (0.6)

SA	PO	0.7	8.1 (0.5)	−1.1 (0.3)	4.7 (1.0)	6.7 (0.4)
		1.0	6.5 (0.6)	−0.2 (0.2)	6.2 (1.8)	5.9 (0.9)
	PC	0.7	−10.0 (0.5)	−2.2 (0.1)	−9.6 (0.5)	10.9 (0.3)
		1.0	−8.5 (1.0)	−2.5 (0.1)	−7.5 (1.0)	9.0 (0.6)

PA	PO	0.7	7.3 (0.7)	−1.3 (0.3)	5.3 (0.7)	6.3 (0.4)
		1.0	6.3 (0.3)	−0.2 (0.2)	6.6 (0.8)	6.0 (0.3)
	PC	0.7	−6.1 (0.4)	−1.4 (0.1)	−4.8 (0.5)	6.0 (0.3)
		1.0	−5.5 (0.4)	−1.4 (0.1)	−4.8 (0.6)	5.5 (0.3)

PEA	PO	0.7	6.2 (0.3)	−1.5 (0.2)	4.2 (1.1)	5.5 (0.4)
		1.0	6.4 (1.1)	−0.4 (0.4)	5.0 (1.0)	5.6 (0.7)
	PC	0.7	−8.5 (0.8)	−1.9 (0.3)	−8.6 (0.8)	9.2 (0.7)
		1.0	−6.5 (0.7)	−2.0 (0.1)	−9.0 (0.7)	8.1 (0.4)

Abbreviations: PA, polishing and anodization; PC, translucent cement; PEA, polishing, etching, and anodization; PO, opaque cement; S, sandblasting; SA, sandblasting and anodization; SD, standard deviation.

**Table 3:** Multiple comparisons of color difference (*ΔE*_00_) between different groups (mean difference, significance, and 95% confidence interval).

Homogenous subsets for *ΔE*_00_ (zirconia/total assembly)									
Cement type PO				Cement type PC					
Surface conditioning	*n*	**Subset**		Surface conditioning	*n*	**S** **u** **b** **s** **e** **t**			
		1	2			1	2	3	4
PEA	24	5.5	—	PA	24	5.7	—	—	—
S	24	5.9	5.9	PEA	24	—	8.6	—	—
PA	24	—	6.1	SA	24	—	—	9.9	—
SA	24	—	6.2	S	24	—	—	—	10.4

Abbreviations: PA, polishing and anodization; PEA, polishing, etching, and anodization; S, sandblasting; SA, sandblasting and anodization.

**Table 4:** Influence of surface conditioning methods, thickness of ZrO_2_ specimens, and the cement type on ΔL^*∗*^, Δa^*∗*^, Δb^*∗*^, and *ΔE*_00_ values.

Measure	Cement type	Zirconia thickness (mm)	Linear regression results			
			*R* ^2^	Constant	Coefficient of regression	
*ΔE*_00_	PO	0.7	0.020	6.59	−0.037	*p* = 0.332
		1.0	0.038	5.29	0.038	*p* = 0.185
	PC	0.7	0.670	4.06	0.374	**p** < 0.001
		1.0	0.636	2.65	0.357	**p** < 0.001

*ΔL* ^*∗*^	PO	0.7	0.007	7.22	0.014	*p* = 0.561
		1.0	0.029	6.35	−0.019	*p* = 0.244
	PC	0.7	0.854	−6.30	0.187	**p** < 0.001
		1.0	0.618	−5.07	0.149	**p** < 0.001

*Δa* ^*∗*^	PO	0.7	0.072	−1.26	−0.009	*p* = 0.065
		1.0	0.025	−0.19	−0.009	*p* = 0.281
	PC	0.7	0.637	−2.09	0.094	**p** < 0.001
		1.0	0.948	−2.28	0.104	**p** < 0.001

*Δb* ^*∗*^	PO	0.7	0.006	4.66	0.006	*p* = 0.595
		1.0	0.039	5.75	0.020	*p* = 0.180
	PC	0.7	0.625	−9.66	0.161	**p** < 0.001
		1.0	0.597	−8.63	0.108	**p** < 0.001

Abbreviations: PC, translucent cement; PO, opaque cement.

For coefficients of regression differing significantly from zero, corresponding *p*-values are highlighted with bold font.

## Data Availability

The source data can be made available upon request.
